# Enhanced Oil Recovery Method Selection for Shale Oil Based on Numerical Simulations

**DOI:** 10.1021/acsomega.1c01779

**Published:** 2021-09-07

**Authors:** Elena Mukhina, Alexander Cheremisin, Lyudmila Khakimova, Alsu Garipova, Ekaterina Dvoretskaya, Maya Zvada, Daria Kalacheva, Konstantin Prochukhan, Anton Kasyanenko, Alexey Cheremisin

**Affiliations:** † Skolkovo Institute of Science and Technology, Center for Hydrocarbon Recovery, Moscow 121205, Russia; ‡ Gazpromneft Science & Technology Centre, St. Petersburg 190000, Russia; § Gazpromneft-tekhnologicheskie partnerstva, St. Petersburg 190000, Russia

## Abstract

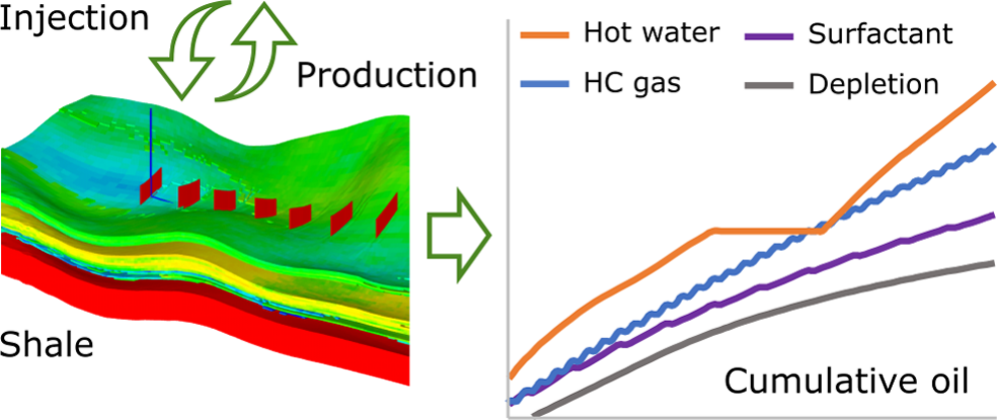

As unconventional reserves, oil shale deposits require additional oil recovery techniques to achieve favorable production levels. The efficiency of a shale reservoir development project is highly dependent on the application of enhanced oil recovery (EOR) techniques. There are many studies devoted to discrete investigations of each EOR method. Most of them claim that one particular method is particularly effective in increasing oil recovery. Despite the wealth of such research, it remains hard to say with certainty which technique would be the most effective when applied in the extraction of unconventional reserves. In this work, we aim to answer this question by means of a comparative study. Three EOR methods were applied and analyzed in the same environment, a single target object—an oil field in Western Siberia characterized by ultra-low permeability (0.03 mD on average) and high organic content. Methods involving huff-and-puff injection of a surfactant solution, hydrocarbon gas, and hot water were studied using numerical reservoir simulations based on preceding laboratory experiments. A single horizontal well having undergone nine-stage hydraulic fracturing was used as the field site model. The comparative calculations of cumulative oil production over an 8-year period revealed that the injection of hot (supercritical) water led to the highest oil recovery in the target shale reservoir. Each EOR method was implemented using the best operation scenario. All three cases resulted in an increase in cumulative oil production compared to the depletion mode, though the efficiency was distinctly different. Twenty-six percent more oil was obtained after hot water injection, 16% after hydrocarbon gas, and 12% after a surfactant solution. Simulation of a hot water huff-and-puff operation over a longer period (43 years) led to a level of oil production 3 times higher than depletion. The drawbacks of each EOR method on the shale site are discussed in the results. A possible solution was proposed for preventing the negative effects of heat loss and water blockage incurred from hot water injection. The comparative study concludes that hot water injection should lead to the highest volume of oil recovery. The conclusions drawn are suggested to be relevant for similar shale fields.

## 1 Introduction

At present, the development of prospective unconventional oil reserves is one of the highest-profile topics in the oil industry. According to various sources, the Bazhenov formation in Western Siberia holds between 65 and 500 × 10^9^ tons of oil in place.^1−3^ The shale reserves within the Bazhenov formation contain light low-sulfur tight oil and a significant amount of solid organic matter—kerogen,^4^ which can adsorb oil, making it more difficult to recover. Today, hydraulic fracturing is the main technology used for oil recovery from ultra-low-permeability reservoirs.

However, only 10% of hydrocarbons (HCs) can potentially be recovered from ultra-low-permeability shale reservoirs using this technique. The low recovery factor is caused by a rapid decline in the recovery rate during the first few years of oil production in depletion mode due to the rapid decrease in reservoir pressure and the limited drainage volume.^5,6^ Moreover, up to 50% of Bazhenov’s organic matter is kerogen, which is capable of *in situ* synthetic oil production only through the application of additional stimulation.^7,8^

Although waterflooding could potentially be effective in low- and ultra-low-permeability reservoirs,^9,10^ other oil recovery agents should lead to a better result, especially for tight reservoirs with mosaic hydrophobization.^11^ Various enhanced oil recovery (EOR) techniques can be applied for the efficient extraction of shale oil. Common screening criteria^12,13^ are not always reliable for unconventional reservoirs. Therefore, each unconventional reservoir requires a special screening test aimed at selecting the most suitable EOR technology for the reservoir’s particular characteristics.

This study aims to investigate the efficiency of EOR methods for developing a single shale field in Western Siberia. The efficiency evaluation was based on forecasting the results of numerical simulations. Some of the simulation parameters were derived subsequent to laboratory experiments. Three EOR techniques were studied with consideration of the technological limitations of developing a single horizontal well after multistage hydraulic fracturing: huff-and-puff injection of hot water, hydrocarbon gas, and a surfactant solution.

The primary mechanisms involved in thermal stimulation are a reduction in viscosity, an alteration in interfacial tension (IFT) and capillary effects, the evaporation of light components, and the eventual increase in oil mobility.^14^ These effects can be significantly boosted by employing catalysts.^15^ Additionally, high temperatures activate the thermal cracking of bitumen and solid kerogen, causing the production of synthetic oil and improving the reservoir’s filtration and capacitive properties.^16,17^ Water is the most feasible heating agent for thermal recovery, as it provides massive heat transfer and activates hydropyrolysis. Moreover, water serves as a catalyst for thermal reactions.^18^ Existing experimental and numerical studies imply that hot water (subcritical or supercritical fluid) injection may potentially lead to a significant increase in oil recovery from oil shale formations due to the increased porosity and permeability and the realization of kerogen’s ability to generate oil^4,19−22^ depending on its type and level of thermal maturity.^23^ It should be noted that hot water is potentially an eco-friendly EOR agent, although well operation should nevertheless be performed very carefully to avoid possible risks.

Although CO_2_ is the most promising EOR gas agent for shales,^24,25^ the lack of source often limits the potential of this method. At the same time, a substantial amount of associated gas is recovered during oil production from shale deposits at the Bazhenov formation. One of the most convenient methods of utilizing this gas is to reinject it back into the reservoir as an EOR agent.^26^ Using hydrocarbon gas as an EOR agent in huff-and-puff mode could be effective in shale deposits^24,27,28^ given the presence of natural fractures.^6,29,30^ The efficiency of this method depends entirely on the minimum miscibility pressure (MMP) at which the associated gas achieves miscibility with the reservoir oil. Miscibility causes oil swelling and a decrease in oil viscosity. Oil swelling leads to a pressure gradient in the matrix, which also enhances oil recovery.^6^ According to laboratory studies on shale samples,^24,31^ the oil recovery factor using miscible gas can be as high as 95%, depending on the injection parameters and the properties of the rock and gas. Field-scale simulations also produce successful results, revealing a 15% increase in oil recovery on average.^6,24,32,33^

The injection of chemical agents does not usually require specialized equipment, which could potentially make this method an economically attractive option for EOR. Surfactants are the most suitable chemicals for increasing oil recovery from shale deposits.^34,35^ The mechanism of enhancing oil recovery by means of surfactant injection involves altering wettability (primarily for shales^36^) and interfacial tension. The decrease in capillary pressure is followed by an increase in the filtration rate and the desorption of hydrocarbons from the rock surface and narrow pore throats. The main constraints on the efficiency of surfactant injection in shale deposits are the ultra-low matrix permeability of the surfactant blend, the adsorption of the surfactant on the surface of the rock, clay swelling, and the surfactant’s instability under reservoir conditions (i.e., the temperature and composition of reservoir water).^37,38^ Therefore, the success of surfactant EOR is dependent upon developing a surfactant composition and concentration suited to the specificities of the particular target shale reservoir. Some of the previously investigated formulations of nonionic and anionic surfactant blends were found to be promising for EOR from particular shale deposits. Experimental spontaneous imbibition studies revealed^24,39^ an increase in oil displacement of up to 60% for even very tight core plugs. An exceptional surfactant huff-and-puff field study^40^ performed for a carbonate shale formation confirmed that surfactant treatment could significantly increase oil production.

All three methods have advantages and disadvantages, which are even more important for an unconventional deposit. This study presents a direct comparison to identify the most promising method of EOR for the development of a shale oil field in Western Siberia.

The success of an EOR technique can be determined from its commercial feasibility, economic assessment, or the amount of oil produced. The conclusions drawn in this study are based on the primary indicator of a development’s efficiency, that is, oil production, according to predictive hydrodynamic simulation studies. Most of the data used in the numerical experiments were obtained from laboratory tests.

## 2 Description and Adjustment of the Simulation Model

### 2.1 Description of the Simulation Model

The target research site was a sector of the Bazhenov formation characterized by ultra-low permeability and high organic matter content around a horizontal well having undergone nine stages of hydraulic fracturing. The geological model of the well with its nine fractures was created based on real field data, including well trajectory and fracture parameters. The single well was drilled to implement huff-and-puff injection technology, which has proven to be the most effective strategy for oil recovery from shales.^24^ The initial reservoir pressure and temperature were 23.4 MPa and 95 °C, respectively. The single-porosity-sector geological model was created using the Schlumberger Petrel software platform. Dual porosity was not applied since no presence of natural fractures was observed in the region. The reservoir model was generated from the results of well logging, seismic data, crosswell correlations, and laboratory research on core samples. A three-dimensional (3D) grid represented the stratigraphic and lithologic boundaries of the formation layers. In all, 38 layers were amalgamated into 7 generalized layers: 5 geological packs of the Bazhenov formation, the lowest layer representing a fractured formation with water saturation below the target range, and a clay interlayer between the Bazhenov formation and the underlying formation. A sequential Gaussian simulation was applied to determine the distribution of most of the properties in each layer, and a sequential indicator simulation was used for the NTG distribution. The simulated model of the reservoir consisted of a total of 252 700 cells with ∼100 000 active cells. Inactive cells present layers that are totally impermeable for the investigated fluids.

**Figure 1:**
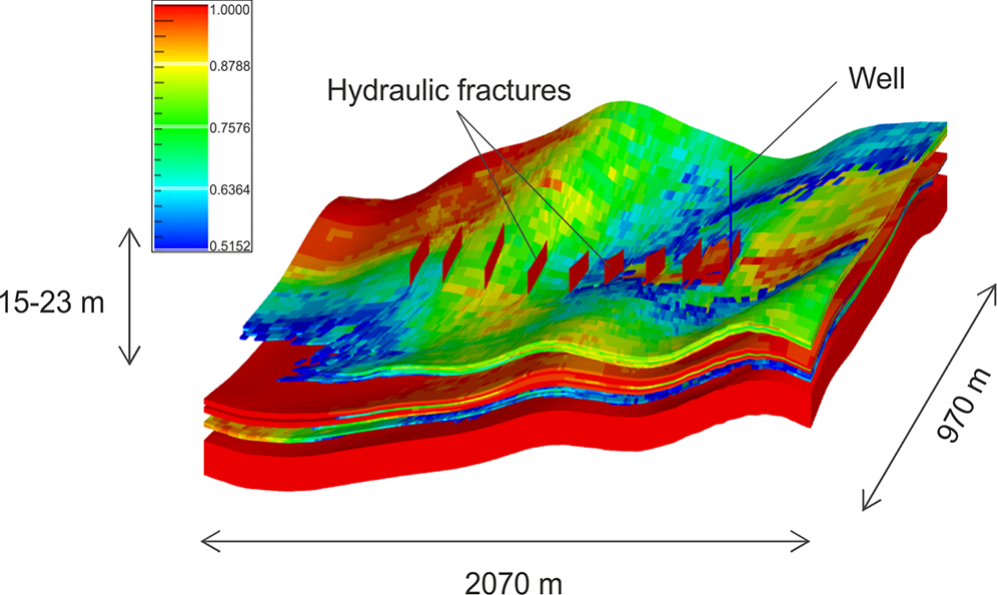
Proportions and NTG distribution in the target sector.

**Figure 2:**
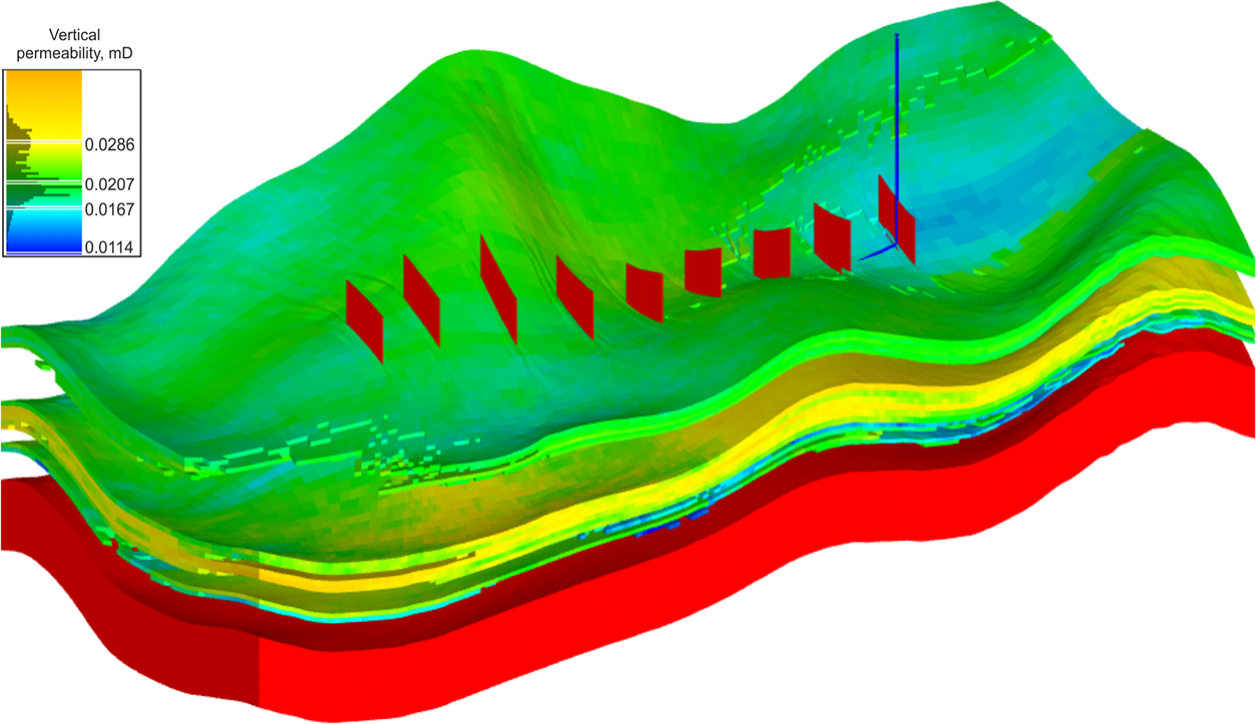
Permeability distribution in the target sector.

The dimensions of the sector were ∼2.1 km (*x*-axis) by ∼1 km (*y*-axis), while the width of the formation varied in a range of 15–23 m (*z*-axis), as illustrated in Figure 1. The total surface area of the sector was ∼1.9 × 10^6^ m^2^. The permeability distribution (Figure 2) was calculated from permeability versus porosity cross-plots. Porosity and permeability were adjusted in correlation with the geological data and laboratory studies of core samples. The average permeability and porosity for different reservoir layers are given in Table 1.

**Table 1:** Average Reservoir Parameters for Different Layers

	pack	range	average
porosity, %	II	6.9–9.5	8.20
	IV	5.1–8.4	6.75
	I, III, V	2.7–3.0	2.85
permeability, mD	II, IV	0.02–0.04	0.030
	I, III, V	0.01–0.04	0.025

**Figure 3:**
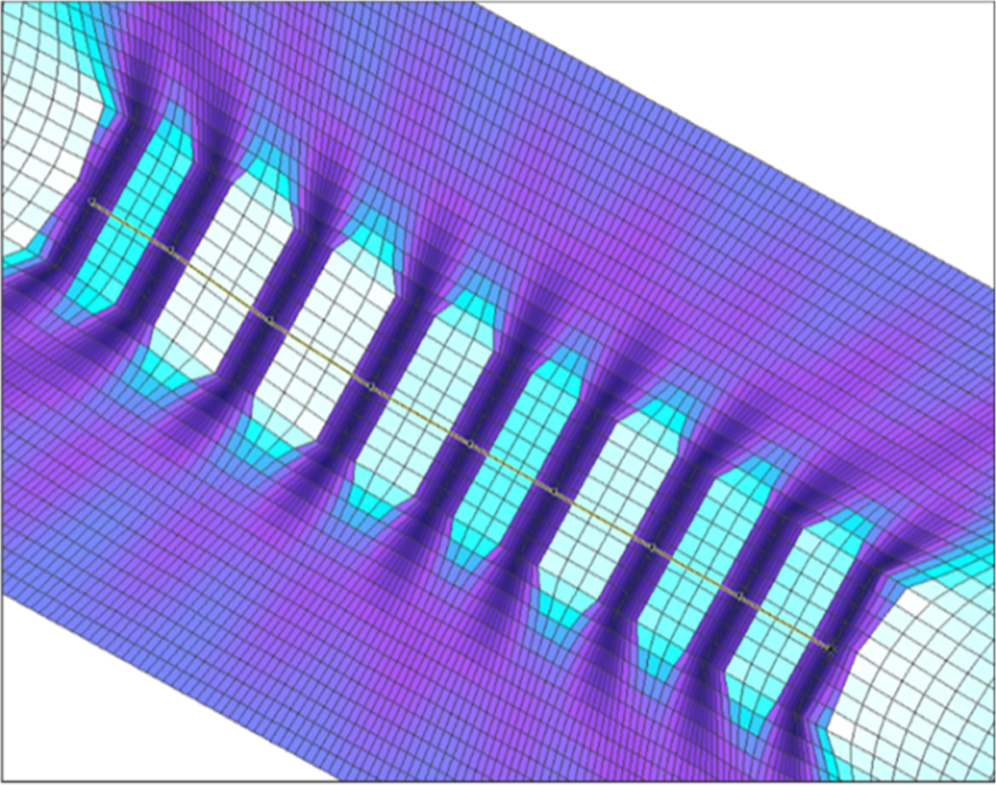
Gradual grid refinement along the *x*-axis.

In the 3D sector model, fracture regions were characterized using nonlinear smooth local grid refinement, allowing the fluid behavior in the fractured zones to be ascertained in detail while avoiding further upscaling. The cell size was increased along the *x*-axis, the greater the distance from the fracture (Figure 3). The simulated fracture width of 1–1.5 m was not enough to represent the physically correct properties of the actual 5–10 mm fractures. Thus, the properties of the fractured zones were scaled by decreasing porosity and increasing permeability. Additional interlayer grids were inserted in every fracture region to provide a vertical connection between the layers.

**Figure 4:**
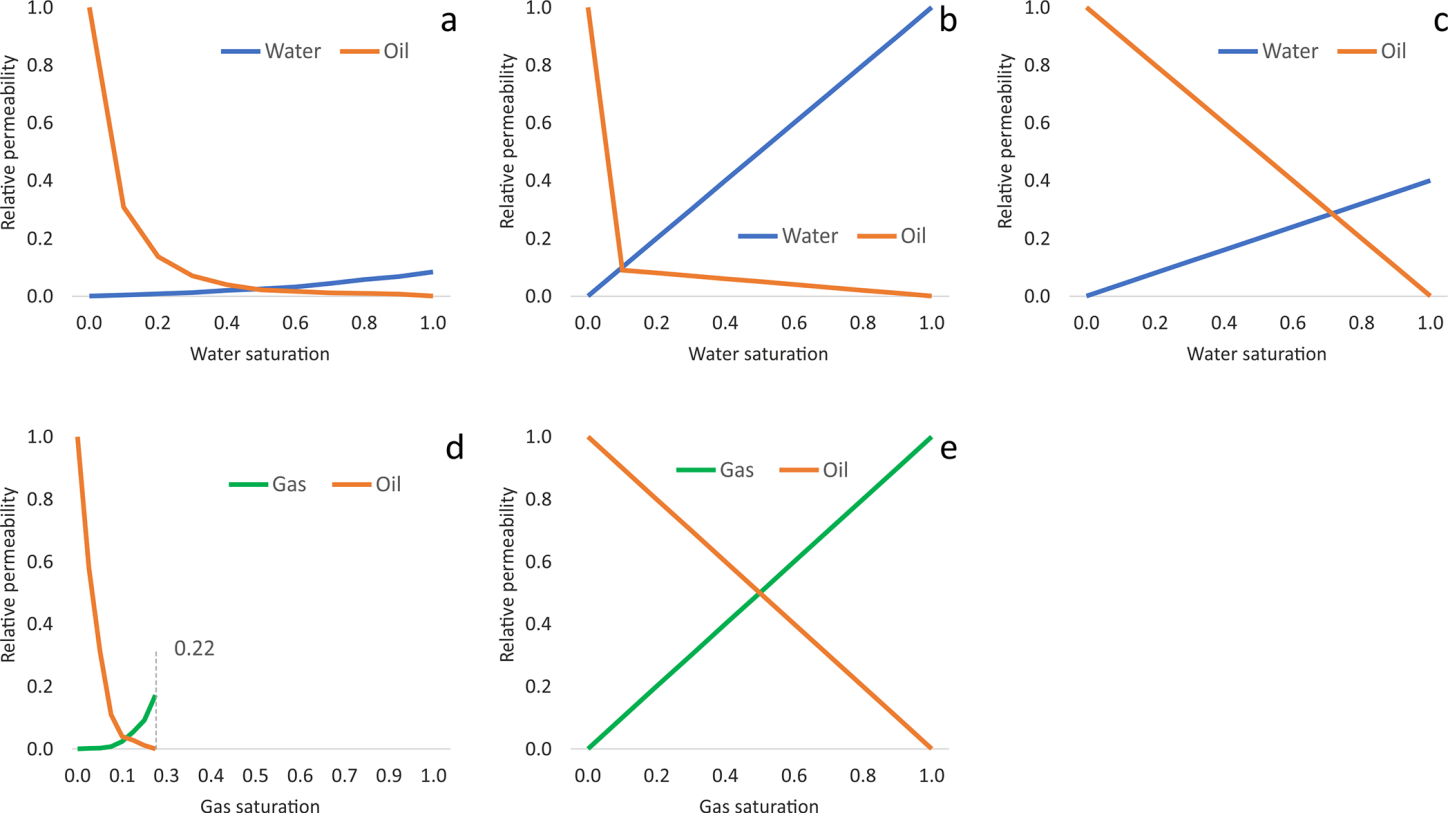
Relative permeabilities of the oil–water system (a—main reservoir, b—underlying formation, c—fractures) and the oil–gas system (d—main reservoir and underlying formation, e—fractures).

Relative-phase permeabilities were specified for three regions—the main reservoir (adjusted after laboratory experiments), the underlying formation with water saturation, and the hydraulic fractures (Figure 4).

**Figure 5:**
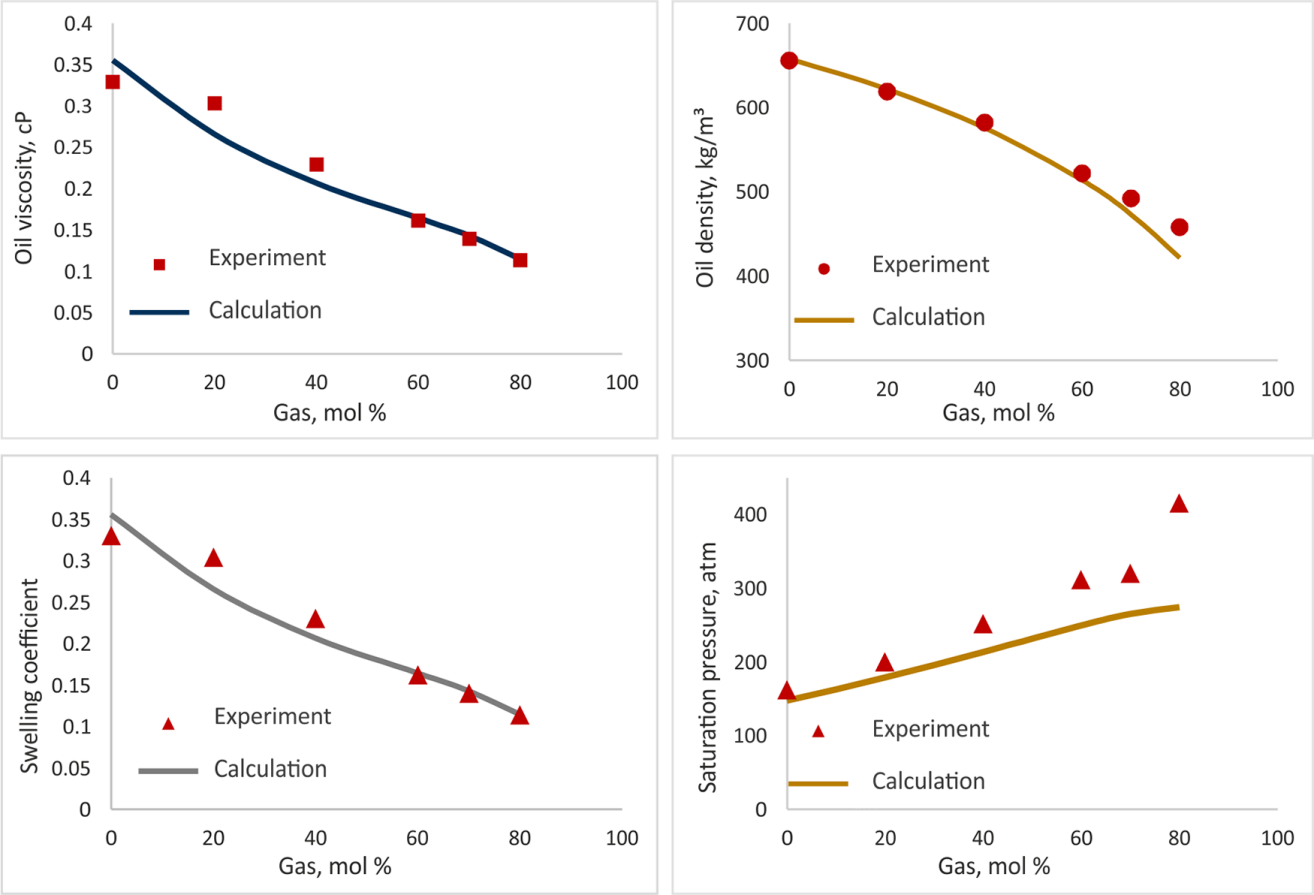
Adjustment of the PVT parameters.

The reservoir’s fluid properties and EoS for compositional and thermal models were calculated in Schlumberger PVTi and CMG WinProp and matched with experimental data (differential and flash liberation, swelling test). The fluid PVT properties and their adjustment are illustrated in Table 2 and Figure 5. The fluid composition used in the reservoir simulations is given in Table 3.

**Table 2:** Results of EoS Adjustment for the Reservoir Oil

parameter	experimental data	simulation	deviation, %
saturation pressure, MPa	13.39	14.05	4.7
oil density at reservoir conditions, kg/m^3^	660.1	673.5	1.9
oil density at standard conditions, kg/m^3^	832	827	–0.6
gas–oil ratio, m^3^/m^3^	145.6	156.4	6.9
formation volume factor	1.556	1.550	–0.3
oil viscosity at reservoir conditions, cP	<0.35	0.39	
oil viscosity at standard conditions, cP	4.88	4.74	–3.0

**Table 3:** Generalized Compositions of Investigated Fluids

	content, mol %	
components	reservoir oil	hydrocarbon gas
CH_4_	34.88	60.34
CO_2_ + N_2_ + + He + H_2_ + C_2_H_6_	10.15	17.58
C_3–4_	13.3	20.48
C_5–7_	10.94	1.60
C_8+_	30.74	0.00
total	100	100

**Figure 6:**
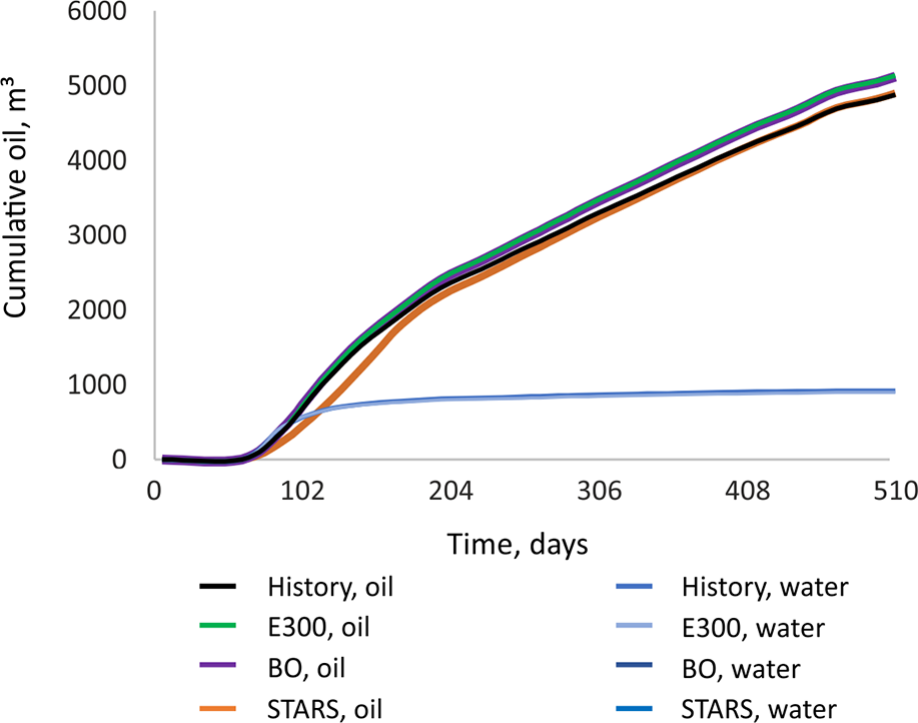
History matching in Schlumberger ECLIPSE 300, tNavigator black-oil, and CMG STARS.

History matching was performed for field production rates, water injection during hydraulic fracturing, and cumulative oil and water production. The matching in cumulative liquid production is shown in Figure 6. Water production matched perfectly—the blue curves cover each other on the graph. Oil production was well-matched with a 5% deviation between simulations.

Numerical simulation of EOR operations was performed using CMG STARS, ECLIPSE 300, and tNavigator with a black-oil fluid. CMG STARS was chosen to model thermal injection as it is an advanced program for modeling thermal reservoir processes.^41^ A compositional simulation in ECLIPSE 300 allowed miscibility processes occurring during hydrocarbon gas injection to be investigated in detail. The black-oil fluid model met all of the requirements for chemical EOR simulation. The history matching in each simulation program was performed separately to achieve similar accuracy.

### 2.2 Adjustments to the Fluid Model for the Simulation of a Hydrocarbon Gas EOR

The oil modeled was based on a generalized composition of reservoir oil (Table 3) and three-parameter Peng–Robinson EoS with a volumetric shift parameter in the program PVTi. The PVT properties were adjusted using multivariate nonlinear regression, and the Lohrenz–Bray–Clark correlation was applied to adjust oil viscosity.

The composition of the target injection gas from Table 3 is the generalized composition of associated gas from the target oil field, which is being investigated as the main candidate for a hydrocarbon gas EOR.

**Figure 7:**
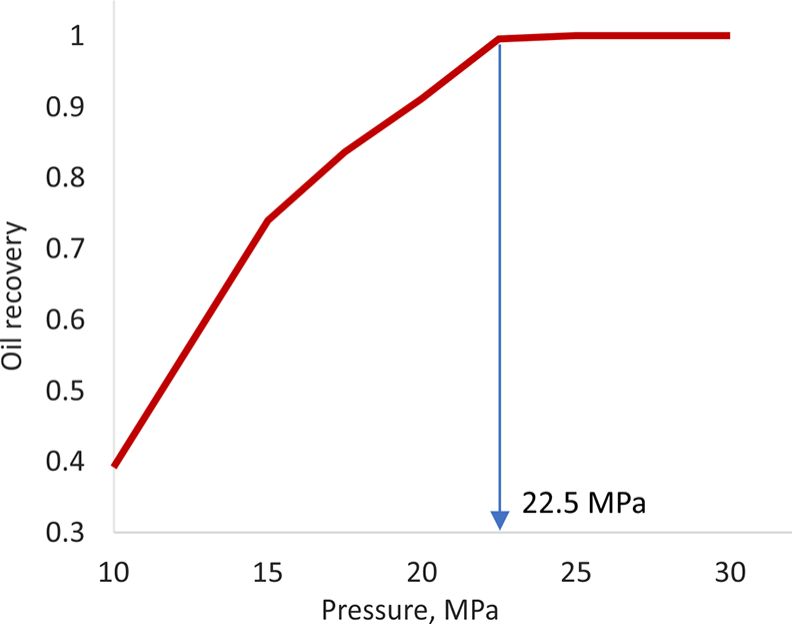
Calculation of MMP using a slim-tube simulation.

A numerical simulation of a slim-tube experiment has been performed to determine MMP for the hydrocarbon gas and the reservoir oil. According to the calculations, within a pressure range of 10–30 MPa, the MMP for the target oil and gas compositions is 22.5 MPa (Figure 7), which is slightly lower than the initial reservoir pressure of 23.4 MPa.

### 2.3 Thermal Pseudo-Compositional Model and Rock Properties for the Hot Water EOR Simulation

The PVT parameters for thermal simulation were set in CMG WINPROP with the following pseudo-components: CO_2_, CH_4_, hydrocarbon gas C_2_–C_5_ (HCG), light oil C_6_–C_18_ (LO), and heavy oil C_19_–C_35_ (HO). The solid phase was represented by bitumen, kerogen, and coke.

Four kinetic reactions were set for simulating the thermal transformation of organic matter.

- Thermal cracking of bitumen, *E*_a_ = 194.2 kJ/mol
  1 mole BITUM = 0.84 mole HO + 0.76 mole LO + 0.76 mole HCG + 13.67 mole COKE.
- Thermal cracking of kerogen, *E*_a_ = 224.1 kJ/mol
  1 mole KER = 2.39 mole HO + 2.16 mole LO + 2.16 mole HCG + 39.05 mole COKE.
- Thermal cracking of HO, *E*_a_ = 230 kJ/mol
  1 mole HO = 0.95 mole LO + 0.95 mole HCG + 17.19 mole COKE.
- Thermal cracking of LO, *E*_a_ = 260 kJ/mol
  1 mole LO = 0.54 mole HCG + 9.68 mole COKE.

The kinetic parameters were calculated based on the Arrhenius equation and the results of a series of laboratory experiments on open-system pyrolysis before and after extraction. The experiments and calculations were performed in accordance with the published model.^42^ The results of the calculations were confirmed through reference to published data on the transformation parameters of kerogen and bitumen.^43,44^

Although these reactions adequately describe the real process of hydrocarbon transformation, future studies must focus on the separate effects of hot water on mobile oil, adsorbed light and heavy oil, viscous bitumen, and kerogen to achieve more reliable calculations.^45^

In addition, the following thermal effects were specified:

- The thermal conductivity of the rock was 1.7014 Wt/(m*K).^46^
- A decrease in residual oil saturation following the increase in temperature (the shapes of the relative permeabilities have an experimentally validated dependence on temperature).
- An increase in effective permeability depending on the increase in porosity after the thermal cracking of solidified bitumen and kerogen and an increase in effective permeability depending on the temperature increase in ultra-low-permeability zones.^47−49^
- The dynamic viscosity was determined by the Andrade equation, which is the most suitable correlation for the temperature range studied.

### 2.4 Simulation Parameters for Surfactant Solution Injection

The target surfactant composition was selected after experimentation with more than 30 blends.^37^ Using a 0.1% solution of anionic and nonionic surfactants, it was possible to identify the best properties for the specific reservoir conditions.

**Figure 8:**
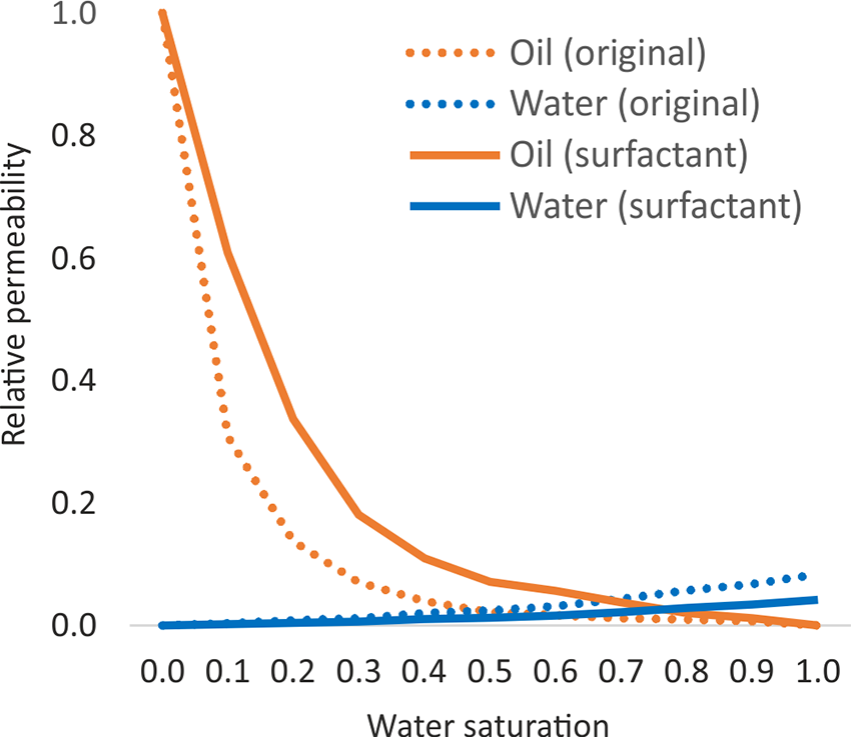
Alteration in oil–water relative permeabilities after adding the surfactant.

The main physical effects in the simulation had previously been laboratory-tested for the chosen solution. The alteration in relative permeabilities for the oil–water system (Figure 8) was based on an observed decrease in oil/water interfacial tension and an alteration in wettability. Residual oil saturation declined due to the weakening of capillary forces and the desorption of the adsorbed oil. The shape of the altered curves was adjusted based in part on an analysis of published results in wettability alteration.^50,51^

Surfactant adsorption on the rock surface was set as a gradual increase in interfacial tension (an adsorption isotherm) due to a decrease in the concentration of the surfactant. The initial concentration of the 0.1% surfactant solution decreased to 0.0092% due to adsorption by 3.632 g/kg of rock. At the same time, IFT increased from 0.0367 to 2.41 mN/m. The desorption process was simulated by the opposite dependence.

In addition, the surfactant concentration affected water viscosity, which was also defined in the simulation.

## 3 Results and Discussion

For the purpose of comparing EOR methods, they should not be applied with identical injection/production parameters. Using identical parameters for different mechanisms will inevitably result in divergent, noncomparable efficiency levels and results. Therefore, to increase the reliability of the study, an optimal EOR scenario was developed for each fluid injected. The following technological parameters of a huff-and-puff process were varied: the pressure and duration of the injection stage, the duration of the soaking stage, and the pressure and duration of the production stage.^52^ Additionally, the temperature of the hot water and the initial concentration of the surfactant solution were determined. The best field development scenario for each EOR case is given in Table 4. In each case, an injection simulation was performed below the fracture initiation pressure (<35 MPa), preventing a breakthrough into the underlying formation.

**Table 4:** Optimal Huff-and-Puff Parameters for the EOR Fluids Investigated

	hydrocarbon gas	surfactant solution	hot water
pressure during injection	30 MPa	33 MPa	30 MPa
duration of an injection stage	1 month	1 month	12 months
duration of a soaking stage	no soaking (variations revealed that a soaking stage of any reasonable duration was not effective in the target field)		1 month
pressure during production	10 MPa	10 MPa	10 MPa
duration of a production stage	1 months	5 months	36 months
characteristic of an injected fluid	5 grouped components	concentration of a surfactant = 0.1%	temperature = 375 °C

The highest volume of oil production could potentially be achieved if injection is initiated while the wellbore pressure is as high as possible.^20^ Since the initial pressure in the target reservoir was relatively low (23.4 MPa), initiation of the injection would ideally occur not long after the well starts operating to avoid significant depletion. At the same time, the water injected during hydraulic fracturing should already be produced back from the fractures and reservoir. Therefore, in each EOR simulation, the injection of agents was started 7 months (∼210 days) after multistage hydraulic fracturing, when water production was practically holding steady without having exhibited any change over a few months, as per the site history (Figure 6).

**Figure 9:**
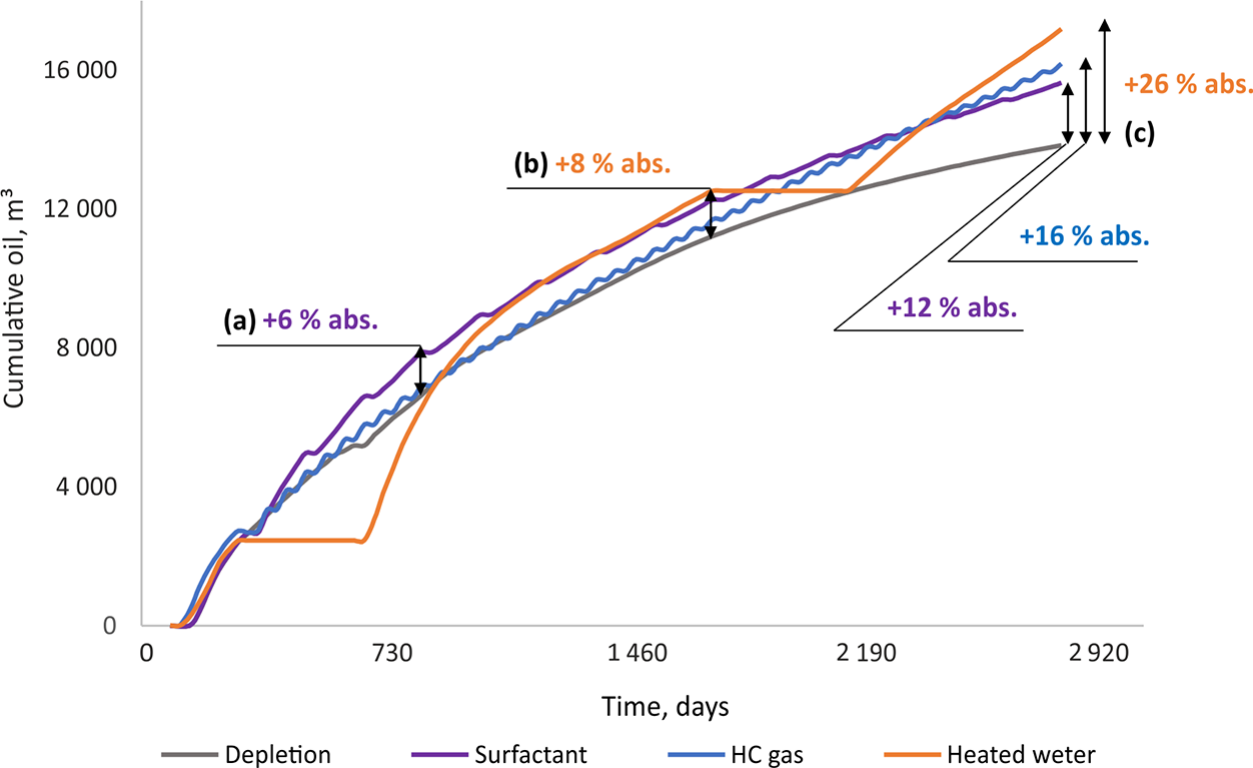
Cumulative oil production in depletion mode and during huff-and-puff EOR operations.

The comparative estimation of EOR efficiency was performed for an 8-year period (Figure 9).

Simulation of the first 7.5 years of the EOR process revealed the following trends (observed in Figure 9):

- During the first 2 years (∼800 days), the most effective EOR technique was the surfactant solution huff-and-puff stimulation, which increased cumulative oil recovery by 6% (an additional ∼1300 sm^3^) compared to depletion mode (a, Figure 9).
- Although the first year of hot water injection was a loss for oil production, the subsequent 3 years (∼1600 days) of the “puff” stage compensated for the loss with an 8% (an additional ∼1600 sm^3^) increase in cumulative oil recovery compared to the depletion mode (b, Figure 9).
- After 6 years (∼2400 days), hydrocarbon gas injection led to a better result than the injection of a surfactant solution.
- Oil production during surfactant and HC gas EOR tends to decline at the end of the 8-year period.
- At the end of the forecast period, the hot water huff-and-puff stimulation becomes the most effective EOR technique, leading to an increase in cumulative oil production by 26%, compared to the 16% from hydrocarbon gas injection and 12% from the surfactant solution (c, Figure 9). Moreover, production did not decline over the 8 years of the well’s operation in hot water huff-and-puff mode. This implies that hot water stimulation could potentially increase oil production even more given longer-term well operation.

**Figure 10:**
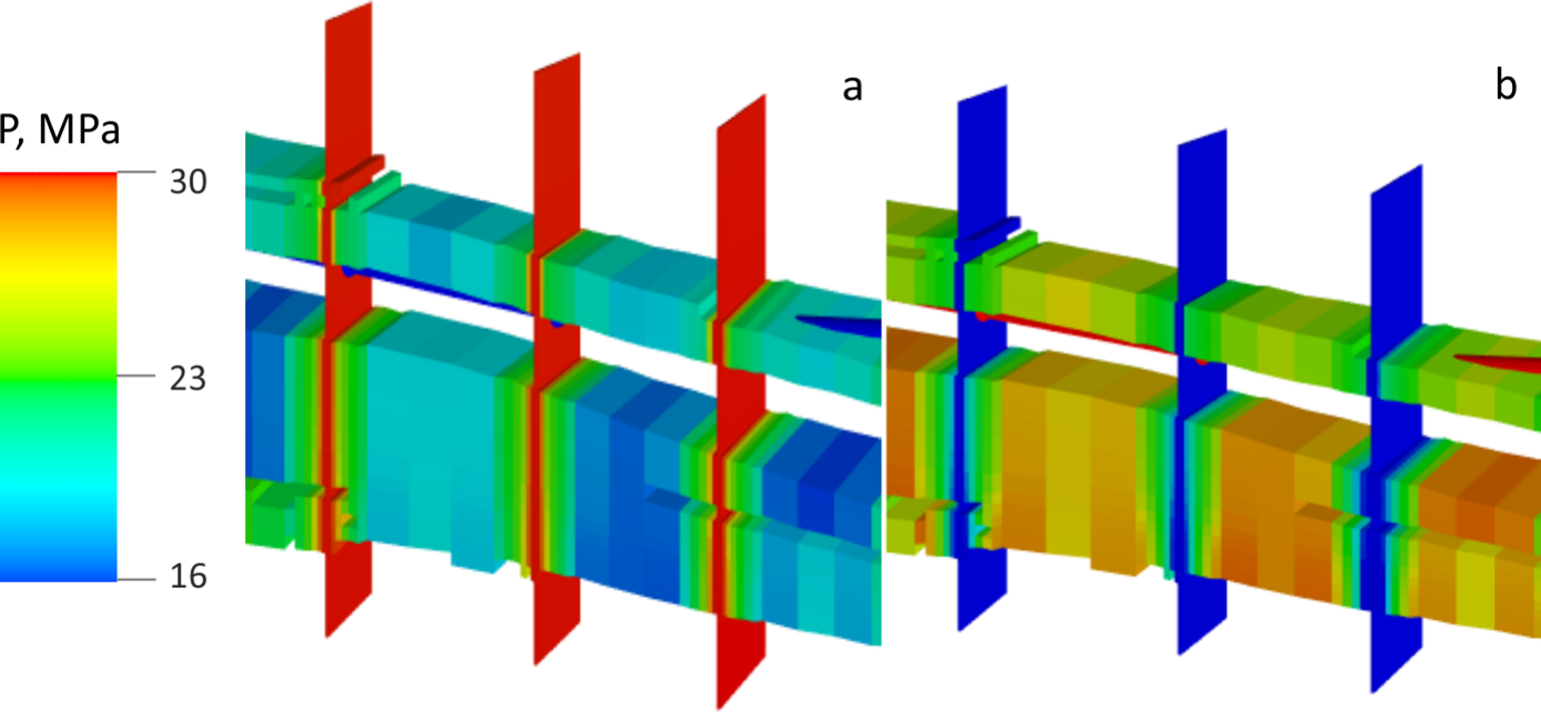
Distribution of pressure during the first hydrocarbon gas “huff” operation (a) and at the end of the last puff operation (b).

During hydrocarbon gas EOR, the gas penetrates into the rock matrix through fractures, with the pressure increasing over the MMP (Figure 10). The decrease in oil viscosity and partial oil swelling allowed additional oil to be recovered from the matrix. However, this effect is perceptible only after 5–6 years of operating the well. The slow increase in the recovery rate could be explained by the reservoir rock’s low relative permeability to gas (Figure 4). The presence of light reservoir oil (Table 3) with low initial viscosity (Table 2) could be another reason for the slow increase in recovery since even miscible gas displacement could not significantly increase the efficiency of extracting this category of oil.

However, the simulation of an HC gas huff-and-puff operation resulted in a recovery of ∼3000 sm^3^ of additional oil over 7.5 years (16% compared to depletion mode, Figure 9), which is a relatively positive outcome. It should be noted that gas EOR would be economically effective only in a well cluster, and not a single well, since the expenses on equipment (compressor, pipelines, etc.) could be recouped only through the profit from several wells.

**Figure 11:**
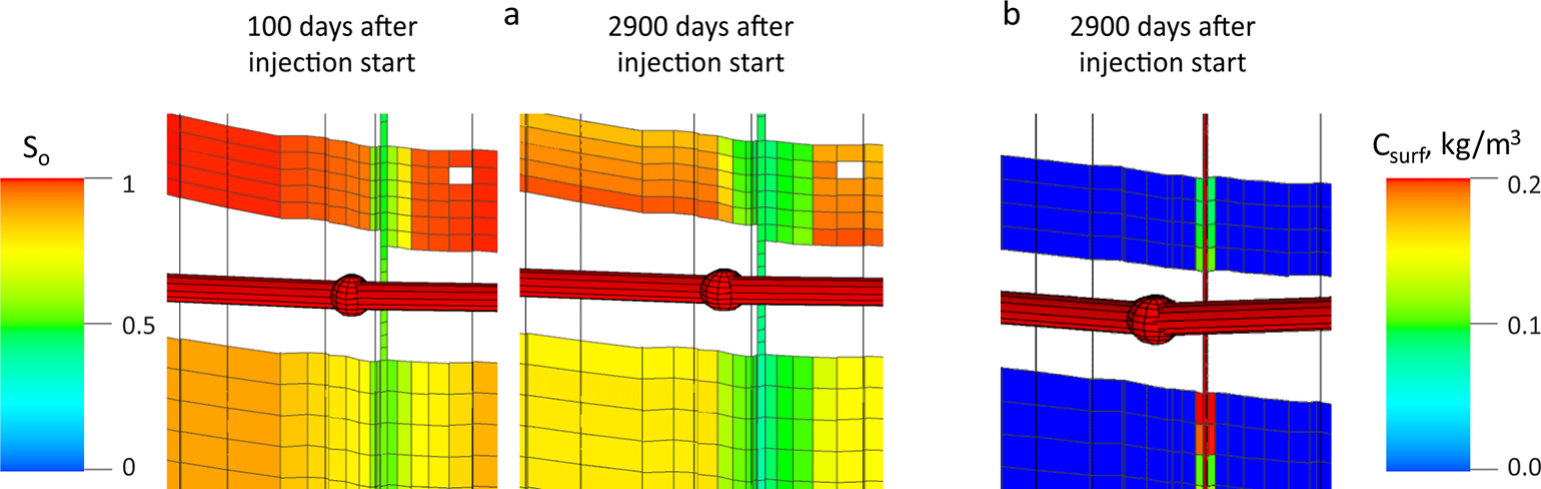
Oil saturation (a) and surfactant concentration at the end of the forecast period (b).

The simulation of a surfactant huff-and-puff operation indicated a slight but fast increase in oil recovery followed by a slow decline in parallel with the depletion curve. Additionally, it was observed that the concentration of the surfactant rapidly decreased while moving away from the fractures. Adsorption prevented the surfactant from further spreading into the rock matrix. However, the pressure increase made it possible to recover oil from zones that are inaccessible to the surfactant (Figure 11).

At the same time, the huff-and-puff surfactant solution operation caused a water blockage near the fractures. Part of the oil was pushed further from the fractures and water saturation was increased in the fracture zones, which eventually countervailed the positive effect.

**Figure 12:**
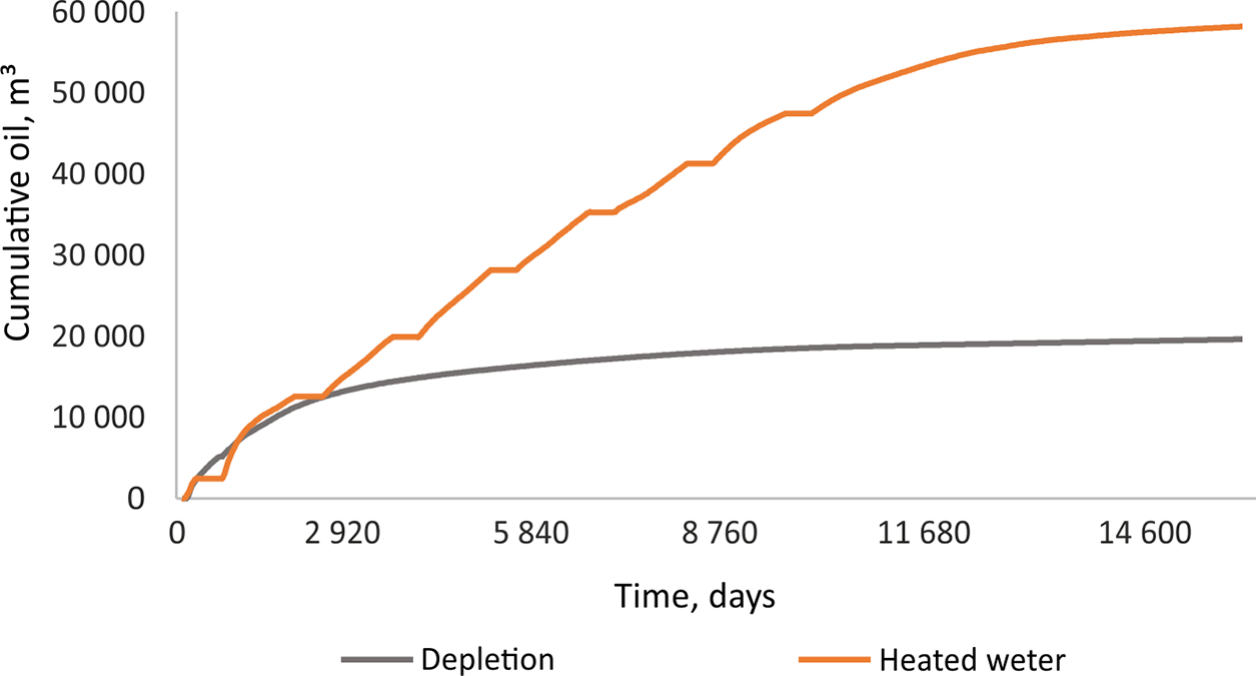
Cumulative oil production during hot water stimulation over ∼43 years.

The most promising result of this study was obtained from the simulation of hot water injection. The positive effect is noticeable after one and a half huff-and-puff cycles (Figure 9) but is even more significant over an extended period. Figure 12 displays the cumulative oil production during continuous hot water EOR operation for ∼43 years and 7 huff-and-puff cycles as compared with the depletion curve. After the seventh injection, the well operated in depletion mode until the end of the forecast period. The simulation results imply that, at the end of the period, oil production should be almost three times higher than depletion production.

According to the calculations performed, over a 43-year period, depletion leads to recovery of 19.6 × 10^3^ sm^3^ of oil, which is only 2.7% of the initial geological reserves. There is the potential to produce 58.2 × 10^3^ sm^3^ of oil, including ∼38.5 × 10^3^ sm^3^ of additional oil using the thermal EOR technique, which would increase the recovery factor to ∼8.3% (Figure 12). In this case, the specific cumulative water consumption would be 0.91 m^3^ per sm^3^ of oil and the specific cumulative thermal energy consumption would be 2.3 GJ per sm^3^ of oil, which is economically viable.

**Figure 13:**
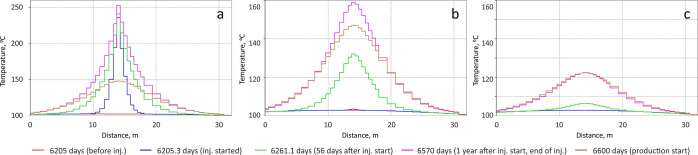
Temperature profiles along a fracture at 0.5 m (a), 1.5 m (b), and 2.5 m (c) during one of the huff stages of hot water stimulation.

**Figure 14:**
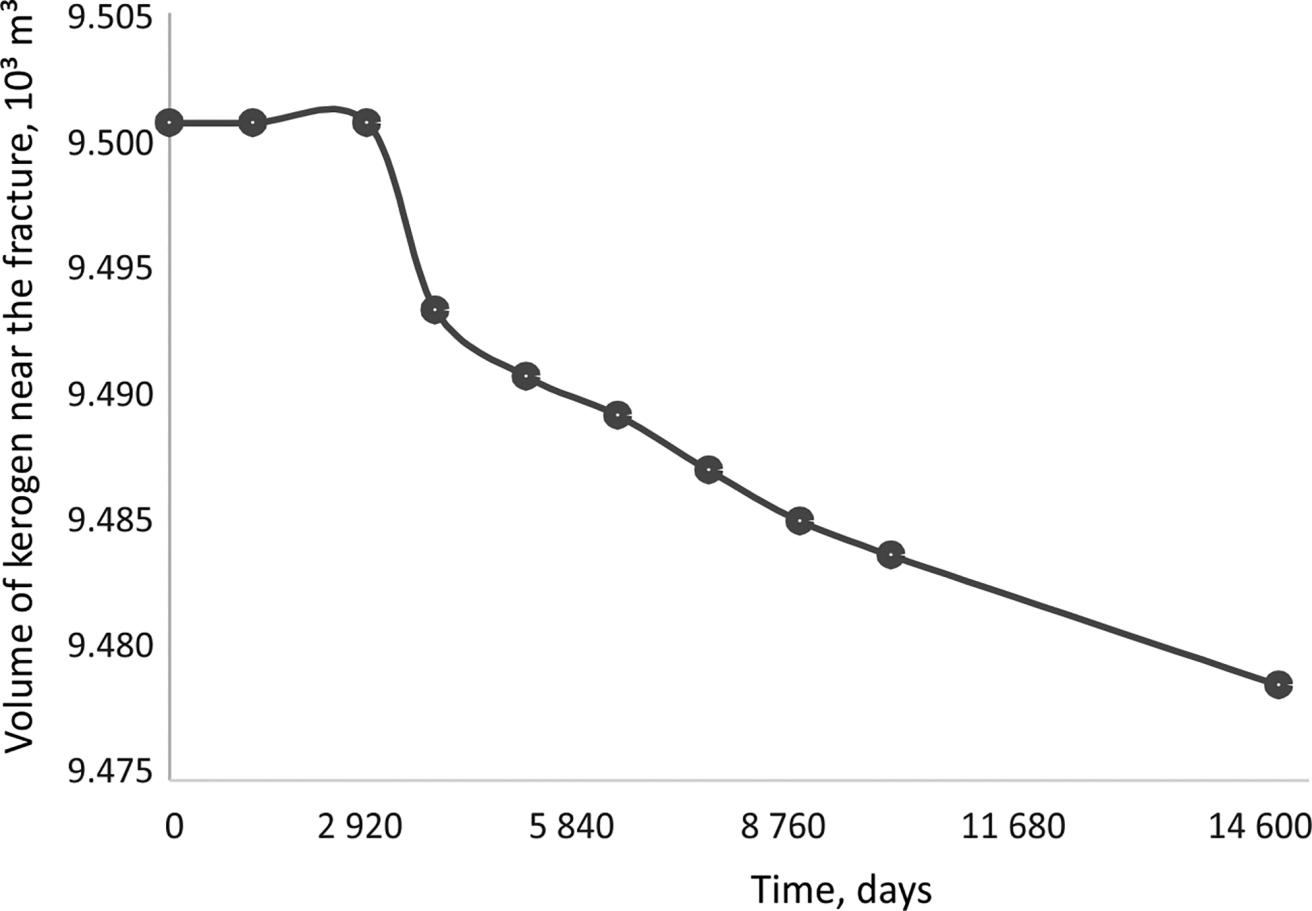
Decrease in the absolute volume of kerogen in the cells closest to the fractures.

Nevertheless, a few problems occurred during the hot water injection process. The insufficient filtration properties of the Bazhenov formation result in a low effective thermal conductivity.^46^ This could lead to poor distribution of temperature in the rock matrix due to heat loss.^20^Figure 13 shows the distribution of heat near a fracture during one of the hot water injection cycles. An effective temperature of >200 °C was maintained at a distance of only 1 m from the fracture. Hence, kerogen transformation and bitumen desorption occurred only near the fractures. However, the transformation of kerogen into “synthetic” oil continued throughout the entire stimulation period. The decrease in the absolute volume of kerogen around the fractures is presented in Figure 14. It should be noted that the transformation of the kerogen began only during the second huff-and-puff cycle.

**Figure 15:**
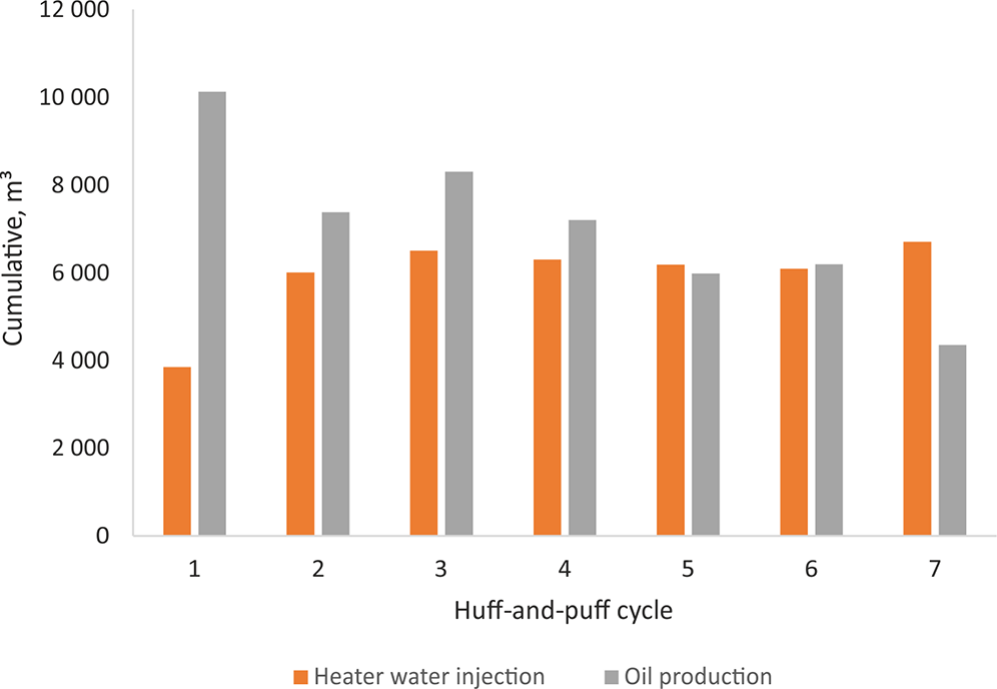
Distribution of cumulative hot water injection and oil production for each EOR cycle.

Just as with surfactant solution injection, the water blockage problem was observed with hot water injection. Figure 15 indicates that oil production falls between the first cycle and the seventh cycle, in direct correlation with the increase in water consumption. The main reasons were an increase in water saturation near the fractures, a reduction in the relative permeability to oil, and a decrease in pressure with every puff operation. However, the massive water blockage is not the only result of a long huff stage. The continuous increase in reservoir pressure is partially driven by thermal pressurization,^20^ which leads to an expanded volume of drained rock.

The heat loss and the water blockage are serious problems for hot water stimulation at the Bazhenov formation, requiring solutions to be found through further research. Despite that, the continuous increase in reservoir pressure, kerogen transformation, and desorption of hydrocarbon components caused by the hot water were enough to result in the highest increase in cumulative oil production.

## 4 Selecting the Most Effective EOR Method and Conclusions

The results of the EOR simulation study for short-term and long-term forecasting imply that the hot water huff-and-puff operation is the most effective EOR method for the target unconventional reservoir. The injection of a heated agent causes a drop in oil viscosity, the desorption of heavy and light hydrocarbon components, the thermal cracking of kerogen, an increase in permeability, and, in addition, a thermally driven increase in reservoir pressure. Thus, the hot water huff-and-puff operation can result in a significant increase in oil recovery, one that is incomparable with other EOR methods. The main mechanisms of thermal EOR invite the proposal that this stimulation technique could also be effective at similar shale oil fields.

On the one hand, gas and surfactant injections are well-developed technologies and could significantly increase the volume of oil produced from the target well. Moreover, despite evincing the best result in terms of numerical simulation, hot water injection entails certain unresolved challenges, which could preclude the level of efficiency attained herein from being achieved in the field.

On the other hand, hot water injection has the greatest potential as an environmentally friendly and safe method of involving kerogen in production and recovering a vast amount of oil, which is the main reason that future studies should focus on solving these problems.

The primary negative effect of hot water injection is brought on by the water’s behavior in the reservoir, including the formation of a massive water blockage and heat loss. These problems do not impede the recovery of oil in high volumes, though they should always be taken into account. There is a potential preventative solution to this problem: the heat loss and water blockage could be partially neutralized by decreasing the distance between the fractures that serve as the injection sources. The desired distance could be achieved by increasing the number of hydraulic fracturing stages. Thereby, the finer and wider fracture network could potentially provide better connections and a more even distribution of heat and water within the rock matrix.

The most controversial issue regarding the hot water EOR is the equipment, which must maintain the injection of an extreme-temperature fluid over a long period of time. The thermal EOR technique is highly promising, so one of the major future goals in the field of shale oil recovery is the development of an optimum design for well construction and hydraulic fracturing tailored to this particular EOR technique.

## Abbreviations

EOR: enhanced oil recovery
EoS: equation of state
BO: black oil
MMP: minimum miscibility pressure
IFT: interfacial tension
HC: hydrocarbon
